# Eosinophilic Granulomatosis With Polyangiitis (EGPA) Hidden in Acute Appendicitis: A Case Revealed Through Histopathological Examination

**DOI:** 10.7759/cureus.88194

**Published:** 2025-07-17

**Authors:** Yoshiyuki Chiba, Naoki Negami, Yasunori Isido, Haruhiko Okada, Hiroyuki Sugo

**Affiliations:** 1 General Surgery, Juntendo University Nerima Hospital, Tokyo, JPN; 2 Gastrointestinal Surgery, Saiseikai Kawaguchi General Hospital, Saitama, JPN

**Keywords:** acute appendicitis, egpa, eosinophilia, histopathological examination, surgical emergency

## Abstract

Eosinophilic granulomatosis with polyangiitis (EGPA) is a rare form of necrotising vasculitis characterised by eosinophilic infiltration and systemic involvement, most commonly affecting the lungs, skin, and peripheral nerves. Gastrointestinal (GI) manifestations are common in patients with EGPA, although histopathological confirmation of GI involvement is infrequent.

Acute appendicitis is a common surgical emergency, but its presentation as an initial manifestation of EGPA is exceptionally uncommon. We report the case of a 54-year-old man with a history of asthma who presented with fever and epigastric pain that later migrated to the right lower quadrant. Laboratory tests revealed leukocytosis and elevated C-reactive protein levels. Abdominal computed tomography (CT) demonstrated mild appendiceal enlargement, and a diagnosis of acute appendicitis was made. The patient underwent laparoscopic appendectomy.

Postoperatively, he developed persistent lower abdominal pain, new-onset bilateral lower extremity pain, and marked eosinophilia (60%). Histopathological examination of the resected appendix revealed dense eosinophilic infiltration and necrotising vasculitis, establishing the diagnosis of ANCA-negative EGPA. This phenotype is known to present with gastrointestinal manifestations and peripheral eosinophilia frequently. He was subsequently transferred to a specialised centre for further treatment.

This case highlights the potential for EGPA to mimic acute appendicitis. When persistent eosinophilia and atypical postoperative symptoms are present, systemic diseases such as EGPA should be considered. Histopathological analysis plays a pivotal role in identifying underlying systemic vasculitis, particularly in cases with unusual clinical presentations. Greater awareness of EGPA as a differential diagnosis in atypical appendicitis may facilitate earlier recognition and timely management.

## Introduction

First described in 1951 by Churg and Strauss, eosinophilic granulomatosis with polyangiitis (EGPA) is challenging to define due to its varied characteristics [1]. The current proposed definition, from the 2012 Chapel Hill Consensus Conference, is based on the presence of eosinophilic tissue infiltration and necrotising small-vessel vasculitis in patients with asthma and eosinophilia [2]. Such events can damage any organ, but classically manifest as pulmonary infiltrates, sinonasal disease, peripheral neuropathy, renal and cardiac involvement, and rashes [3]. EGPA is generally classified into two immunophenotypes based on the presence or absence of antineutrophil cytoplasmic antibodies (ANCA). ANCA-positive cases are typically associated with vasculitic features such as glomerulonephritis and mononeuritis multiplex, while ANCA-negative cases more often exhibit eosinophilic (allergic) manifestations, including pulmonary and gastrointestinal involvement [2]. Gastrointestinal symptoms occur in about 50% of EGPA cases; however, few reports show histologic evidence of GI involvement. The pathology is difficult to document, but the small bowel is most commonly affected, followed by the stomach and colon [4].

Acute appendicitis is a common surgical emergency that is typically caused by luminal obstruction due to factors such as fecaliths, lymphoid hyperplasia, and neoplasms [5]. While appendicitis is a well-studied condition, it is rare for a systemic disease such as vasculitis to be the underlying cause. There are only a few case reports of acute appendicitis associated with systemic vasculitis, and cases of EGPA presenting as acute appendicitis are particularly rare.

Here, we report a case of ANCA-negative EGPA diagnosed by histopathological examination of an appendix resected for suspected acute appendicitis. This case underscores the importance of considering systemic diseases in the differential diagnosis of acute appendicitis, particularly in the presence of atypical clinical features or unexpected histopathological findings. By reporting this unique presentation, we aim to raise awareness among clinicians about the potential for systemic diseases to mimic common surgical conditions and emphasise the diagnostic value of histopathological evaluation in identifying underlying systemic vasculitis.

## Case presentation

A 54-year-old man with a history of asthma presented to the emergency department of our hospital with a three-day history of fever and epigastric pain, which had gradually migrated to the right lower quadrant (RLQ). At a local clinic, laboratory tests revealed elevated inflammatory markers, and physical examination showed RLQ tenderness and muscular guarding. Acute appendicitis was suspected, and the patient was referred to our facility for further evaluation and treatment.

**Figure 1:**
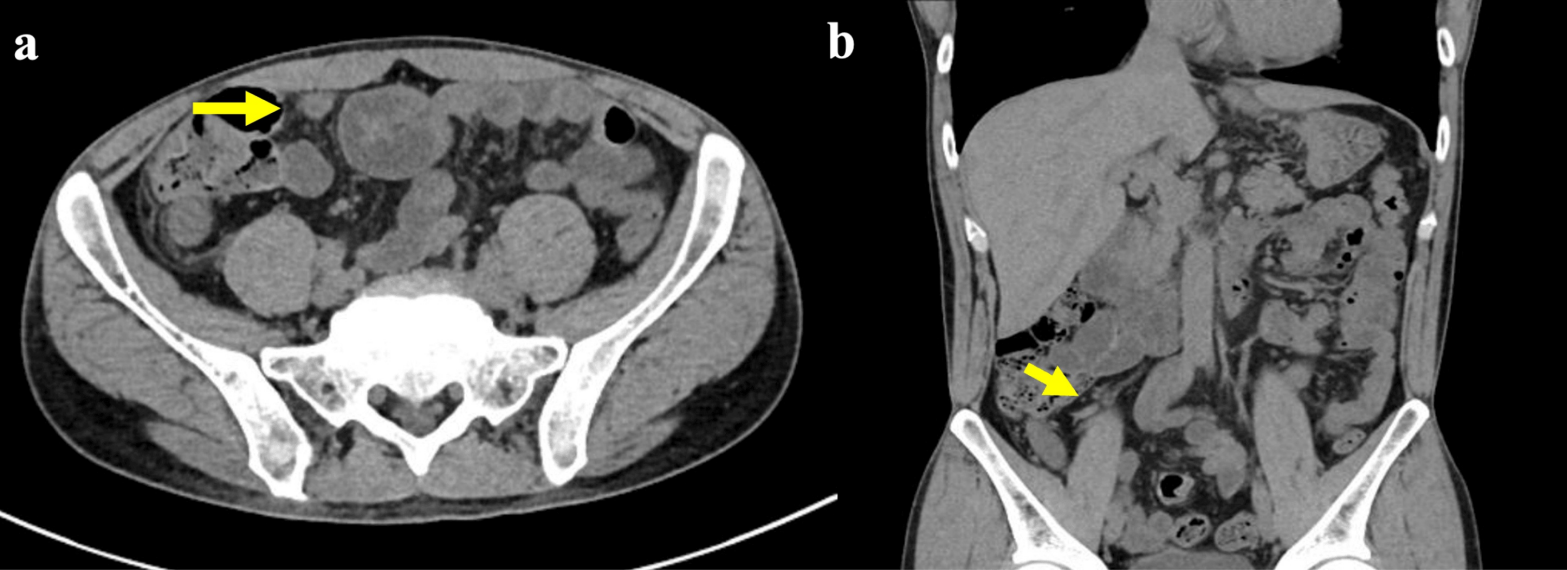
Abdominal computed tomography showing mild enlargement of the appendix. (a) Axial view (b) Coronal view.

On arrival, his vital signs were stable. Abdominal examination revealed RLQ tenderness, muscular guarding, and rebound tenderness. Laboratory tests showed leukocytosis (white blood cell (WBC) count: 19,100/μL) and elevated C-reactive protein (CRP: 3.72 mg/dL). Abdominal CT demonstrated mild enlargement of the appendix without evidence of perforation or abscess formation (Figures 1a, 1b).

**Figure 2:**
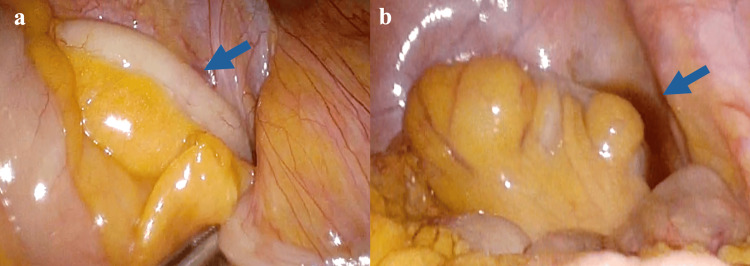
Intraoperative findings. (a) The appendix was adherent to the cecum and mildly enlarged (b) Ascitic fluid was observed in the pelvic cavity.

Based on these findings, acute appendicitis was diagnosed, and laparoscopic appendectomy was performed on the same day. During surgery, the appendix was found to be adherent to the cecum and mildly enlarged, with ascitic fluid observed in the pelvic cavity (Figures 2a, 2b).

**Figure 3:**
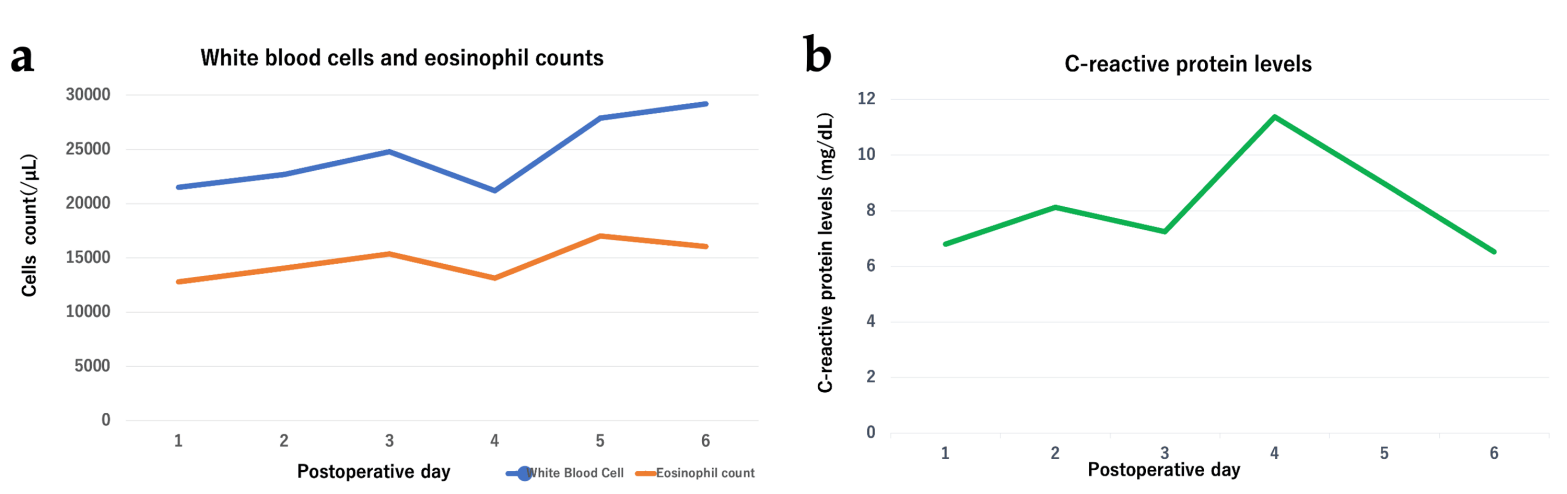
Postoperative changes in inflammatory markers. (a) White blood cell and eosinophil counts (b) C-reactive protein levels.

After careful dissection, the mesoappendix and appendiceal artery were ligated, and the base of the appendix was secured and removed. Postoperatively, the patient reported persistent lower abdominal pain and newly developed bilateral lower limb pain. Postoperative laboratory tests demonstrated a further elevation in C-reactive protein along with peripheral eosinophilia markedly exceeding the normal range (100-500/μL) (Figures 3a, 3b).

Antineutrophil cytoplasmic antibody (ANCA) testing was negative. Histopathological examination of the resected appendix revealed infiltration of eosinophils and neutrophils, consistent with phlegmonous appendicitis. However, due to the persistence of symptoms-particularly bilateral lower limb pain suggestive of peripheral neuropathy, marked eosinophilia, a re-evaluation of the initial diagnosis was undertaken. Although electromyography (EMG) was not performed, the distribution and nature of the pain raised suspicion for peripheral nerve involvement secondary to a systemic condition.

Detailed history-taking revealed a background of otitis media, chronic sinusitis, and allergic dermatitis. Based on these findings and the atypical clinical course, eosinophilic enteritis and eosinophilic granulomatosis with polyangiitis (EGPA) were considered in the differential diagnosis.

**Figure 4:**
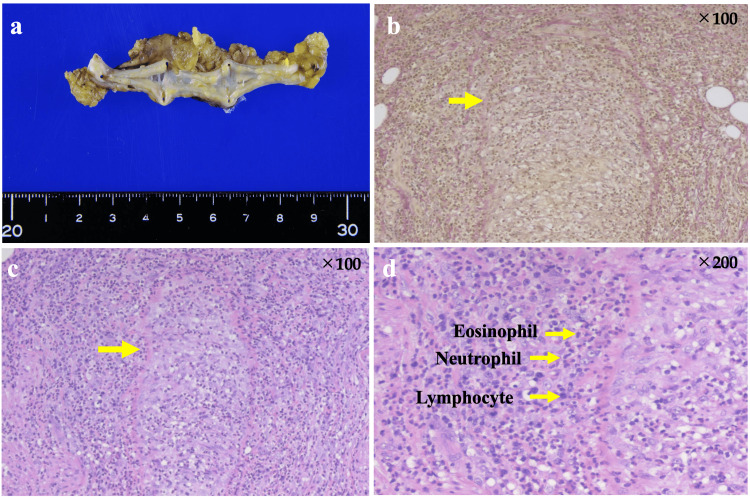
Histopathological findings for the appendix. (a) In the appendiceal specimen, the mucosa appeared atrophic on gross examination (b) Blood vessel walls stained red with Elastica Van Gieson staining (c) Hematoxylin and eosin staining of the appendiceal artery revealed fibrinoid necrosis of the vascular walls. Dense infiltration of inflammatory cells and histiocytes was observed, leading to luminal obstruction (d) An enlarged view  showing perivascular infiltration of eosinophils and neutrophils.

A multidisciplinary discussion with pathologists led to further histopathological investigations. In the appendiceal specimen, the mucosa appeared atrophic on gross examination (Figure 4a). Elastica Van Gieson staining revealed red-stained vascular walls, indicative of collagen fiber fibrosis (Figure 4b). These findings suggested the possibility of an immune-mediated vascular process. Prominent infiltration of eosinophils, plasma cells, and lymphocytes in the walls of medium- and small-sized submucosal vessels was observed, along with fibrinoid necrosis characteristic of necrotising vasculitis (Figures 4c, 4d).

**Figure 5:**
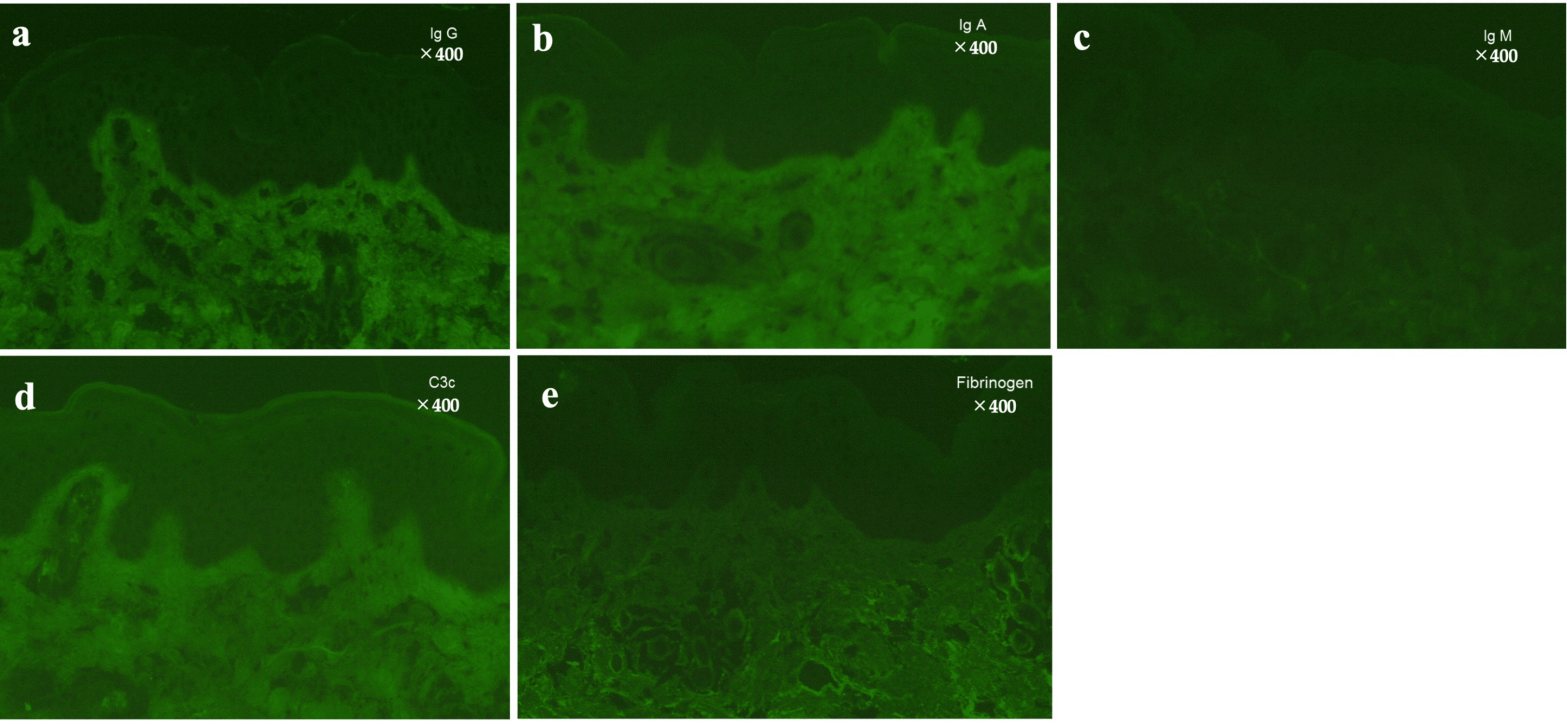
Immunofluorescence staining. (a) IgG staining (X400): Negative, (b) IgA staining (X400): Negative, (c) IgM staining (X400): Negative, (d) C3c staining (X400): Negative, (e) Fibrinogen staining (X400): Negative. All images demonstrate the absence of immune complex deposition. All of the staining tests were negative.

Fluorescence staining of the appendix was negative, indicating the absence of ANCA (Figures 5a-5e).

The history of bronchial asthma, otitis media, sinusitis, and allergic dermatitis, along with postoperative peripheral eosinophilia (60%), bilateral lower limb pain suggestive of peripheral neuropathy, and histopathological evidence of necrotising vasculitis, led to a final diagnosis of ANCA-negative EGPA. Based on all of the findings, we concluded that appendicitis was a secondary manifestation of inflammation caused by EGPA. On postoperative day 7, the patient was transferred to a specialised facility for further treatment.

## Discussion

The etiology of EGPA is unknown, but it may be related to environmental, genetic, and immune factors. The pathogenesis is believed to be mainly ANCA-mediated vascular wall damage and eosinophil infiltration-mediated damage. ANCA can activate neutrophils to generate reactive oxygen species and release lysosomal proteolytic enzymes, which can affect the function of vascular endothelial cells and increase vascular permeability. Clinically, 30% to 50% of patients with EGPA are ANCA-positive, with myeloperoxidase (MPO)-ANCA accounting for 71.4% to 100% of ANCA-positive cases. Two main immunophenotypes can be distinguished based on the presence or absence of ANCA, with ANCA-positive cases associated with vasculitis features and ANCA-negative cases showing allergic manifestations [6].

Vasculitis features such as glomerulonephritis, peripheral neuropathy, and purpura occur more often in ANCA-positive cases, whereas so-called eosinophilic features such as cardiac involvement and gastroenteritis are more frequent in ANCA-negative cases. Asthma and ear-nose-throat (ENT) disease occur in >90% and 60-80% of EGPA cases, respectively, with an equal distribution in ANCA-positive and ANCA-negative cases [7]. Of all systemic manifestations, GI involvement is a negative prognostic factor, especially in cases with mesenteric infarction and bowel perforation. GI involvement occurs in about 50% of patients with a diagnosis of EGPA, with symptoms including abdominal pain, vomiting, and diarrhoea. However, histologic evidence for GI involvement is limited, and the pathology is difficult to document, although the small bowel is most commonly affected, followed by the stomach and colon [4].

Diagnosis of EGPA is guided by criteria proposed by the American College of Rheumatology (ACR). These criteria stipulate that a diagnosis can be made in cases with at least four of the following six features: asthma, peripheral blood eosinophilia (eosinophils >10% of the total leukocyte count), a history of atopy or allergic disease, non-fixed pulmonary infiltrates, paranasal sinus abnormalities, and histological evidence of extravascular eosinophil infiltration. Although classified as a vasculitis, the affected tissue usually does not show necrotising vasculitis or granulomata, but rather non-destructive infiltration of the vessel walls by eosinophils [8]. The typical pathological features of EGPA include eosinophilic tissue infiltration of blood vessel walls, which may be accompanied by granulomata and evidence of eosinophilic vasculitis. Histopathological evidence of vasculitis is more common in ANCA-positive cases than in ANCA-negative cases. EGPA lesions often display a combination of eosinophilic infiltration (with or without granuloma formation) and necrotising vasculitis, making it difficult to clearly distinguish whether the lesions are primarily eosinophilic or vasculitic in origin [7,9].

EGPA initially presenting as acute appendicitis is rare, but several case reports have described similar presentations. One notable case involved eosinophilic appendicitis with concurrent gastric perforation in a patient with EGPA, with the diagnosis confirmed histologically following appendectomy and gastric repair [10].

In the present case, the patient initially presented with symptoms mimicking acute appendicitis, leading to an emergency appendectomy. However, the persistence of postoperative symptoms, including bilateral lower limb pain, progressive eosinophilia, and a negative ANCA test, raised suspicion of an underlying systemic disease. Histopathological findings of eosinophilic infiltration and necrotising vasculitis further supported the diagnosis of ANCA-negative EGPA. Thus, this case shows that EGPA can present with gastrointestinal symptoms, and histopathological examination becomes crucial for diagnosis when eosinophilia and atypical inflammatory findings are present. Clinically, EGPA is often associated with a history of asthma, peripheral neuropathy, sinusitis, and skin lesions, as observed in our case. These findings reinforced the suspicion of EGPA as the underlying cause of the symptoms.

As shown in this case, eosinophilic granulomatosis with polyangiitis (EGPA) can present with a wide range of clinical manifestations and may occasionally mimic common surgical conditions such as acute appendicitis. Therefore, in patients with atypical features of acute appendicitis-particularly those with peripheral eosinophilia-systemic diseases such as EGPA should be included in the differential diagnosis.

EGPA is a rare form of systemic vasculitis with heterogeneous and often nonspecific clinical manifestations involving multiple organ systems, which makes timely and accurate diagnosis particularly challenging. A comprehensive diagnostic approach that includes clinical assessment, laboratory testing, imaging, and histopathological evaluation is essential [11]. Due to its multisystemic nature, diagnosis and treatment frequently require a multidisciplinary team approach [12].

EGPA typically responds well to immunosuppressive therapy. Therefore, early clinical suspicion and prompt diagnosis are critical to improving patient outcomes [13]. This case highlights the importance of histopathological examination in confirming EGPA in patients presenting with atypical gastrointestinal symptoms and peripheral eosinophilia.

## Conclusions

This case shows the potential for EGPA to present as acute appendicitis, a rare phenomenon. While acute appendicitis is typically caused by luminal obstruction, clinicians should consider systemic diseases, such as vasculitis, when encountering atypical presentations or persistent postoperative symptoms. Histopathological examination played a pivotal role in establishing the diagnosis, as it revealed eosinophilic infiltration and necrotising vasculitis in the resected appendix. The presence of marked peripheral eosinophilia, lower limb pain, and a history of asthma and allergic conditions further supported the diagnosis of ANCA-negative EGPA. Given that EGPA can mimic common surgical emergencies, heightened clinical awareness is essential. This highlights the importance of thorough histopathological assessment in identifying systemic diseases that mimic surgical emergencies.
